# A Study of the Effects of Different Indoor Lighting Environments on Computer Work Fatigue

**DOI:** 10.3390/ijerph19116866

**Published:** 2022-06-03

**Authors:** Yuan Fang, Chang Liu, Chengcheng Zhao, Hongyu Zhang, Weizhen Wang, Nianyu Zou

**Affiliations:** 1 School of Engineering Practice and Innovation-Entrepreneurship Education, Dalian Polytechnic University, Dalian 116034, China; 2 School of Information Science and Engineering, Dalian Polytechnic University, Dalian 116034, China; liuchanglcc@yeah.net (C.L.); chengzhao31@gmail.com (C.Z.); universezhang0427@163.com (H.Z.); n_y_zou@dlpu.edu.cn (N.Z.); 3 Human Factors and Intelligent Design Research Center, Dalian Polytechnic University, Dalian 116034, China; wz-wang@foxmail.com

**Keywords:** indoor lighting environment, fatigue, EEG, ECG, HRV

## Abstract

The indoor lighting environment is a key factor affecting human health and safety. In particular, people have been forced to study or work more for long periods of time at home due to the COVID-19 pandemic. In this study, we investigate the influence of physical indoor environmental factors, correlated color temperature (CCT), and illumination on computer work fatigue. We conducted a within-subject experiment consisting of a 10 min-long task test under two different illumination settings (300 lx and 500 lx) and two CCTs (3000 K and 4000 K). Physiological signals, such as electroencephalogram (EEG), electrocardiograph (ECG), and eye movement, were monitored during the test to objectively measure fatigue. The subjective fatigue of eight participants was evaluated based on a questionnaire conducted after completing the test. The error rate of the task test was taken as the key factor representing the working performance. Through the analysis of the subjective and objective results, computer work fatigue was found to be significantly impacted by changes in the lighting environment, where human fatigue was negatively correlated with illumination and CCT. Improving the illumination and CCT of the work environment, within the scope of this study, helped to decrease the fatigue degree—that is, the fatigue degree was the lowest under the 4000 K + 500 lx environment, while it was relatively high at 3000 K + 300 lx. Under indoor environment conditions, the CCT factor was found to have the greatest effect on computer work fatigue, followed by illumination. The presented results are expected to be a valuable reference for improving the satisfaction associated with the lighting environment and to serve as guidance for researchers and reviewers conducting similar research.

## 1. Introduction

Work fatigue is a suboptimal psychophysical condition, caused by continuous work [1]. The associated symptoms usually include poor eyesight; blurred vision; and, in severe cases, brain fatigue and headaches. With the increasing popularity of computers and the diversification of software functions, computer work has become the most effective form of work in this era. Computer vision syndrome (CVS) describes a group of eye- and vision-related problems, such as vertigo and dry eyes, resulting from prolonged computer use [2]. The major factors associated with CVS are either environmental (improper lighting, display position, and viewing distance) and/or dependent on the user’s visual abilities [3]. Nearly 60 million people suffer from CVS globally, especially undergraduate students [4], resulting in reduced productivity at work and reduced quality of life of computer workers [5]. However, visual fatigue and brain fatigue caused by prolonged computer use can severely the impair cognitive functions of users, reducing the worker’s operating ability and work accuracy [6]. Therefore, computer work fatigue detection has become a research hot spot in the fields of driving and workplace ergonomics.

As an indispensable part of the indoor environment, artificial lighting largely affects the physiology and psychology of individuals, such as their circadian rhythm, human performance, emotion, and cognition [7,8]. The constancy of the luminous flux in the light environment, and the associated flickering effect, play a crucial role in visual and brain fatigue. Studies have shown that the adverse physiological reactions caused by stroboscopic flicker include distraction and vision loss [9]. With the popularization of photobiological effects theory [10], the role of nonvisual effects in the evaluation of lighting environments has gained more attention. Zhonggui-Yin et al. [11] conducted a clinical analysis of 503 cases of asthenopia and found that the lighting environment had a greater impact on asthenopia. Soo Young Kim et al. [12] showed that fluctuations in work illumination can affect the copywriting of office workers, as well as visual inspection by VDT-related staff. Llinares et al. [13] have investigated the effect of light on student performance and showed that, while attention improves with higher light levels, memory improves with lower light levels. Furthermore, higher CCTs generated better performance in both attention and memory tasks. Additionally, different lighting environments have different effects on visual fatigue. Mingwei-Xu et al. [14] found that the visual fatigue of operators increased as the light intensity increased under various illumination environments (400 lx, 550 lx, 700 lx). Zhang Rui et al. [15] demonstrated that people under high illumination at 1000 lx are more inclined to sustain attention, and 300 lx + 4000 K was recommended for university architectures. MG Figueiro et al. [16] demonstrated that lighting systems delivering a circadian stimulus (CS) value ≥0.3 can reduce sleepiness and increase alertness in office workers. Cajochen et al. [17] indicated that high-intensity light can increase alertness and reduce drowsiness compared to low illumination. Banu Manav [18] demonstrated a preference for a 4000 K color temperature, compared to 2700 K, for impressions of “comfort and spaciousness” in an office.In his study, the participants liked “the combined color temperature mood” and the majority considered using it in their offices.

Fatigue quantification methods can be mainly divided into four categories: subjective scale methods, objective experiment methods, observation methods, and physiological index measurement methods [19]. A subjective scale method requires subjects to evaluate their fatigue through a questionnaire. By using a subjective evaluation method, Mingli Lu et al. [20] concluded that illumination has a very substantial effect on the perception of fatigue and relaxation. Rachmawati et al. [21] used questionnaires for a fatigue rating scale, based on work fatigue, to describe the overall work fatigue assessment. Physiological signal measurement methods can provide a realistic indication of fatigue, mainly through monitoring physiological parameters (e.g., EEG, ECG, Eye Movements). Y Wang et al. [22] studied the visual fatigue associated with VDT work, and showed that physiological signals have a high correlation with subjective visual fatigue. The rhythm waves in EEG, such as low-frequency waves (θ waves and α waves) and high-frequency waves (β waves), can effectively reflect changes in fatigue and alertness [23,24,25]. The ratio of different frequency waves in the EEG can also be used for the assessment of fatigue. Zhang et al. [26] found that the parameters β/α and (α + θ)/β can be used to measure fatigue. ECG is also a reliable technique for detecting fatigue. Even simple recording of changes in heart rate can be used as an indicator of fatigue detection. Takahashi et al. [27] used a multiple linear regression model to assess driver fatigue and concluded that the three indicators of the ECG signal (i.e., HR, HF, and RMSSD) showed remarkable changes as the level of driving fatigue increased. Physiological monitoring methods based on eye movements have the advantage of noninvasiveness. Studies have shown that pupil diameter, saccade speed, and fixation number are all associated with the development of work fatigue. [28]. Hu Xinyun et al. [29] overloaded subjects for an hour and found that the blink duration, average saccade speed, and saccade duration all increased, while the diameter of the pupil decreased.

In this study, we explore the relationship between different lighting environments and computer work fatigue through a combination of subjective, behavioral, and physiological aspects. Subjectively, the fatigue degree is quantified through a fatigue self-test questionnaire. Behaviorally, a task test is performed, and the degree of work fatigue is represented by task performance (error rate). Physiologically, the degree of fatigue under each light environment is estimated by collecting changes in EEG, ECG, and Eye Movements.

Although studies based on the relationship between human fatigue and the illuminated environment have become popular, the underlying functions remain unclear, as most research has been conducted in laboratory settings using inappropriate instruments. In particular, multifactorial analyses remain rare, due to the combination of various physiological indicators.

## 2. Experimental Methodology

### 2.1. Environmental Setup

#### 2.1.1. Space

**Figure 1:**
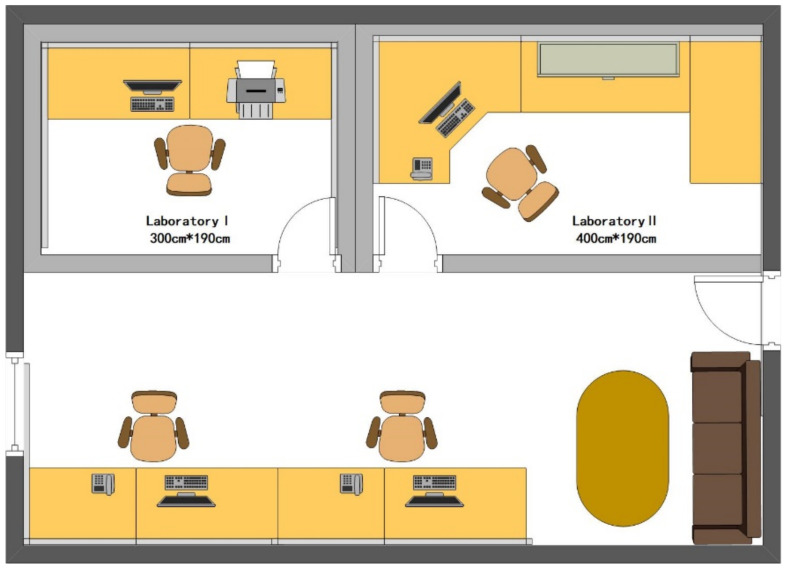
Detailed view of full-scale mock-up model space.

The experiment was performed in a full-scale mock-up office space, with dimensions of 210 cm-wide, 190 cm-deep, and 300 cm-high. Figure 1 shows the detailed layout of the mock-up space. There were no windows in the room to prevent natural light from interfering with the experiments. Two desks with dimensions of 1.2 m (L) × 0.45 m (W) × 0.75 m (H) were placed in the space.

To prevent the influence of external light on the experiment, the inner walls of space were covered with black cloth to avoid any specular reflection. Considering that there were obvious gradient changes of lighting factors in the environmental setting, glare conditions generated by the position of the light source were avoided as much as possible in order to create a good visual environment for the subject. Therefore, the vertical distance between the lamp stand and the desktop and the horizontal distance between the subject and the computer screen in this experiment was no less than 60 cm. The lighting equipment included Philips T5 bracket lamps, which meet the needs of indoor lighting. A desktop computer was installed to perform the test tasks. The visual display terminal adopted an IPS (In-Plane Switching)-type LCD panel, ensuring the stability of the picture and preventing the flicker effect. In addition, it had an ultra-low radiation value, about 0.11 μT. The specific parameters of the experimental equipment are provided in Table 1.

LED tubes were placed on a shelf at an appropriate distance. Increasing or decreasing the number of tubes was conducted to meet the required illumination or correlated color temperature (CCT) levels for the experiment. During the experiment, various measures were taken, such as measuring the light environment parameters in real-time and turning off the automatic brightness adjustment function of the display terminal in order to maintain the constancy of the luminous flux, reduce the flicker effect, and avoid affecting the experimental results.

#### 2.1.2. Working Conditions

The architectural lighting design standard GB 50034-2013 stipulates that the color temperature of ordinary offices is required to be in the range of 3300–5300 K, with the standard illumination value of 300 lx. Considering the photobiological safety of LEDs, the color temperature of LED light sources should be no more than 4000 K.

In order to avoid the influence of human thermal sensation differences in the experiment, it was necessary to ensure that the subjects were in the thermal comfort range. In [30], human thermal comfort has been discussed for Cyber-Physical Human-Centric Systems in smart homes. In [31], the correlation of personal lighting comfort model factors have been analyzed in Cyber-Physical Human-Centric Systems.

This experiment was carried out in winter. The Royal Chartered Building Equipment Association (CBBSE) [32] has provided indoor environmental design specifications to meet thermal comfort, which recommend that the suitable temperature for an office in winter is 20–22 ${}^{\circ }$C, and the humidity should be less than 50%. According to the Chinese standard GB 50019-2003 [33], the standard temperature for comfortable working in winter should be in the range of 20–24 ${}^{\circ }$C, and the relative humidity should be in the range of 30–70%. Generally, when the temperature is 16–25 ${}^{\circ }$C and the relative humidity is 30–70%, there is little effect on the thermal sensation of the human body. According to the standard GB/T18049-2000, the Predicted Mean Vote (PMV) value of the evaluation index characterizing the human thermal response (heat and cold) should be −1⩽ PMV ⩽+1. Based on the requirements of the above specifications for lighting environment and thermal comfort, we designed four different lighting environments. Parameters of the specific operating conditions are given in Table 2.

### 2.2. Subjects

We screened eight subjects who met the requirements of the experiment. Four males and four females with normal color vision and no eye disease participated. The experiment required the subjects to work and rest normally in the 24 h before the experiment, in order to ensure that they had received adequate sleep. No stimulating drinks, such as alcohol, coffee, or functional drinks, were consumed 5 h before the experiment. No kind of physical or mental exercise was performed 3 h before the experiment. The ISO7730, issued by the International Organization for Standardization (ISO), stipulates that the applicable conditions of the comfort standard are as follows: The person is sitting, engaged in light physical activity (Metabolic Rate M < 1.2 MET), and the clothing insulation is 0.5 clo in summer and 1.0 clo in winter. In this experiment, the clothing insulation of the subject was set to 0.9–1.04 clo, which basically met the international standard, and a metabolic level of 1.0 MET was assumed. In addition, the thermal insulation of the seat selected in the experiment can be ignored. Subject information is provided in Table 3.

### 2.3. Data Acquisition Method

#### 2.3.1. Physiological Methodology

1. EEG
  An electroencephalograph (EEG) is the sum of the spontaneous electrical activity of pyramidal cell dendrites on the cerebral cortex, which can effectively reflect brain fatigue and the state of cerebral cortex excitement [34]. Therefore, in this study, EEG signals were selected as evaluation indicators reflecting the effects of different lighting environment parameters on fatigue.
2. HRV
  Heart rate variability (HRV) is a set of quantitative indicators reflecting the activity of the autonomic nervous system. It is an effective indicator of the body’s mental and physical fatigue and workload [35].
3. Eye Movement
  The human eye is the organ that perceives light most directly. Data related to changes in eye activity, such as pupil diameter, number of fixation points, and distribution area, can intuitively reflect the fatigue state.

#### 2.3.2. Subjective Methodology

Subjective methods used in this study included a subjective fatigue scale and task evaluation. The subjective scale method requires subjects to answer questions in the table according to their own perceived state. This method provides a visual representation of the subject’s true state [36].

#### 2.3.3. Metering Operation Method

The metering operation method is a commonly used method for testing work efficiency. Moreover, work efficiency is an important indicator that reflects the visual effect. Common visual task scales include letter recognition, image recognition, and so on. For this experiment, we adopted the original material design. Through analysis of the test error rate of the subjects, their work efficiency can be judged, reflecting the fatigue degree of computer work under the various lighting environments [36].

#### 2.3.4. Equipment

In the experiment, a 16-channel EEG cap was used to collect EEG, where the EEG signal acquisition method was semidry electrode measurement. A physiological information collection multimodule was used to collect the ECG signal. The use of an eye tracker is common for eye movement recording. It is able to record eye movement information while, at the same time, overlaying eye movement information with scene images, allowing for comparison of the experimental data with the real scene. The specific parameters of the physiological signal acquisition equipment are given in Table 4.

#### 2.3.5. Analysis Platform

For data collection and processing, we mainly used the Ergo-LAB human–machine environment synchronization test cloud platform, which has been widely used for experimental design, data collection, analysis and statistics, and human behavioral research.

### 2.4. Experimental Materials

#### 2.4.1. Task Test

**Figure 2:**
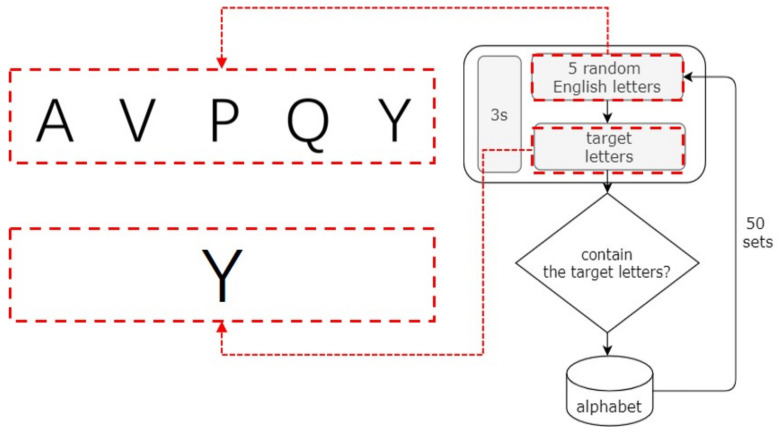
Premise of letter discrimination test.

**Figure 3:**
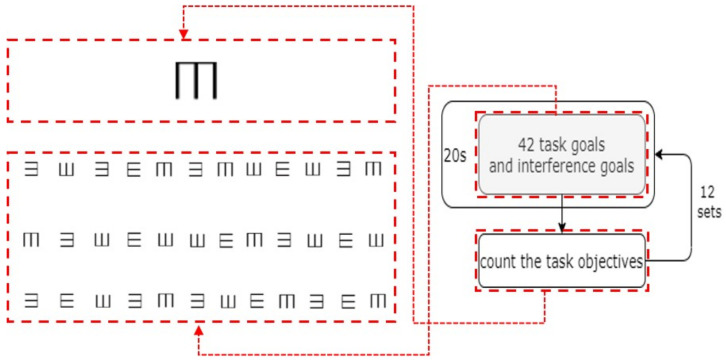
Contents of E-word memory test.

- Letter Distinguishment Test
  The letter distinguishment test used random English letters and a target letter. The subject was required to judge whether the target letter was included in five random letters within the specified time, as shown in Figure 2. In such a test, the participant’s mental resources may be quickly exhausted, due to the constant refreshment of their short-term memory, thus providing an indication of their fatigue state.
- E-word Memory Test
  The E-word memory test, shown in Figure 3, requires the subject to count the number of target items out of a given combination of target and interference items. All of the target items that were wrongly selected, selected multiple times, or missed were judged as wrong. In this test, the intensity of daily computer work can be simulated by eliminating interference and maintaining short-term memory.

#### 2.4.2. Subjective Fatigue Questionnaire

**Figure 4:**
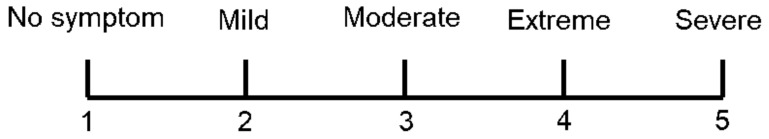
Fatigue evaluation scale.

The subjective fatigue questionnaire contained eight fatigue status items, including vertigo, headache, and so on. Subjects provided scores for these items individually, where a higher score represented a deeper fatigue state. The evaluation scale is shown in Figure 4.

### 2.5. Experimental Process

**Figure 5:**
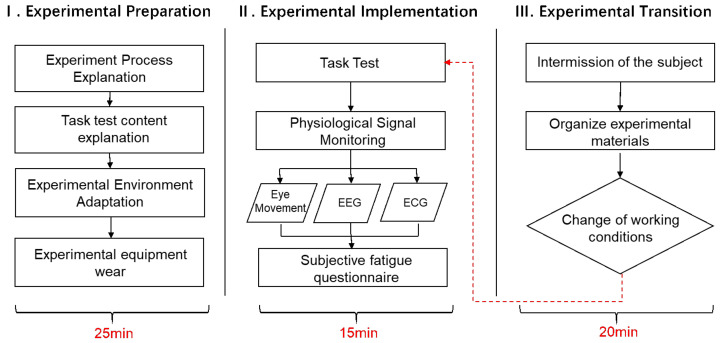
Experimental flowchart.

The experimental process was divided into three parts: Experimental Preparation, Experimental Implementation, and Experimental Transition. The experimental process is depicted in Figure 5.

1. The experimenters and participants first carried out relevant preparatory work before the experiment. The preparation process included an explanation of the experiment process and an introduction to the experimental equipment.
2. Participants performed the task test for 10 min. After that, the subjects were required to fill in a questionnaire according to their subjective feelings towards the lighting environment.
3. After a 10 min intermission, the subjects entered different working conditions for the next round of the experiment.

## 3. Results and Analysis

### 3.1. Subjective Questionnaire

**Figure 6:**
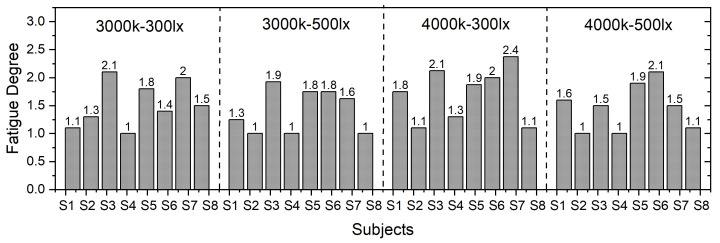
Subjective fatigue under different lighting environments.

The average value of the eight subjective evaluation indices was calculated as a fatigue index for each subject under different working conditions, as shown in Figure 6.

The fatigue index size distribution under different working conditions was relatively uniform. However, subjective fatigue was lower under C3-I5 (3000 k + 500 lx) and C4-I5 (4000 k + 500 lx). It can be speculated that the low color temperature gave the subjects a sense of comfort, while the high color temperature and high illumination led to a bright feeling. However, the subjective data showed great interpersonal differences; so, further analysis of objective data is required.

### 3.2. EEG

EEG has high time resolution and excellent characteristics in the frequency domain. According to the physiological characteristics of EEG in different frequency bands, it can be divided into α, β, γ, δ, and θ waves [37]. The EEG frequency classification is shown in Table 5.

**Figure 7:**
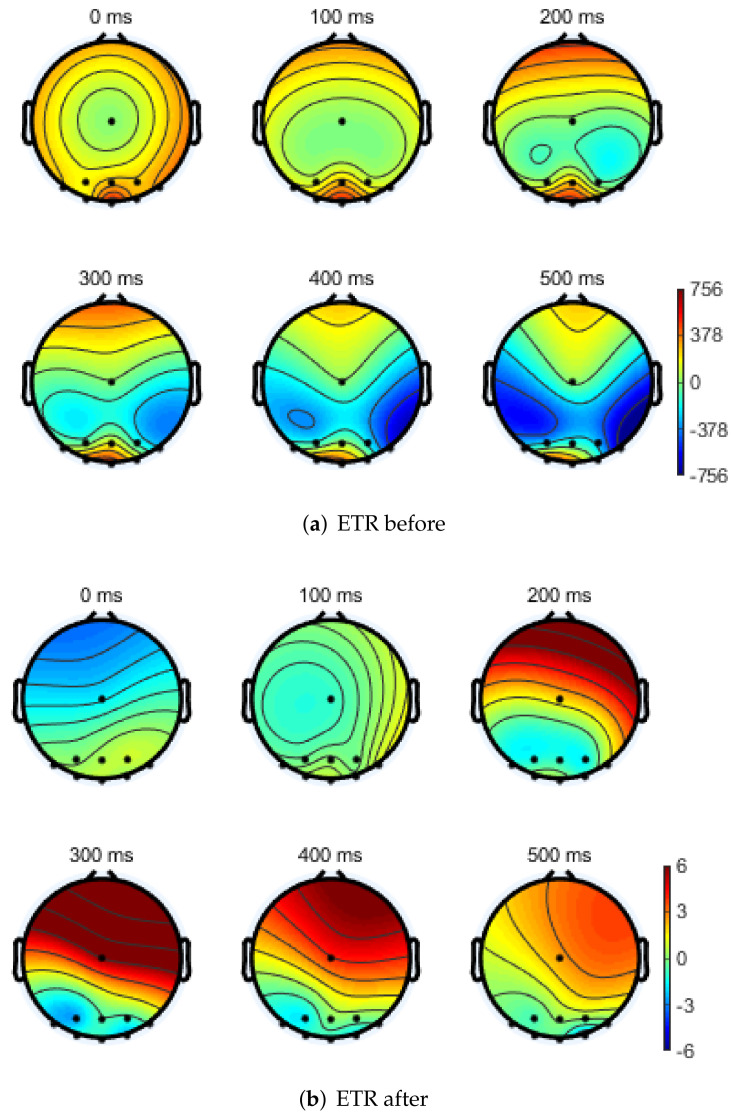
EEG topographical representation.

Electroencephalography topographical representation (ETR) can reflect changes in brain function and express color intensity changes; using this, a percentage topographic map of the ratio between different brain wave frequency band combinations can be obtained. In this experiment, the ETR of the subject at rest was compared with that after the task test. As shown in Figure 7, the change in state after the task test had a significant effect on the energy distribution of the different rhythm waves in the EEG. We further analyzed the proportion of rhythmic waves and the proportion of fast and slow waves to measure the degree of fatigue.

Studies have shown that, when the cerebral cortex is inhibited, the slow-wave content gradually increases in the fatigue state and the θ wave appears [38]. As stated in [39], an increase in θ and δ waves indicates a serious degree of brain fatigue. The characteristic parameter of the basic rhythm of EEG, the Frequency Band Energy Ratio (FBER, hereafter referred to as *R*), represents the proportion of δ, θ, α, and β waves in the total wave. The magnitude and change of *R* can be used to effectively judge the degree of brain fatigue. The frequency band energy ratio *R* was obtained using Equations (1) and (2) as follows:

(1) $$E_{all}\left(k\right)=\sum E^{\left(j\right)}{\left(k\right)}_{j},$$

(2) $$R=E^{\left(j\right)}\left(k\right)/E_{all}\left(k\right),$$

where

*j* is any frequency band of δ, θ, α, and β waves (db);
$E^{\left(j\right)}\left(k\right)$ is the power value of the frequency band (db);
$E_{all}\left(k\right)$ represents the total power value of the four bands (db).

**Figure 8:**
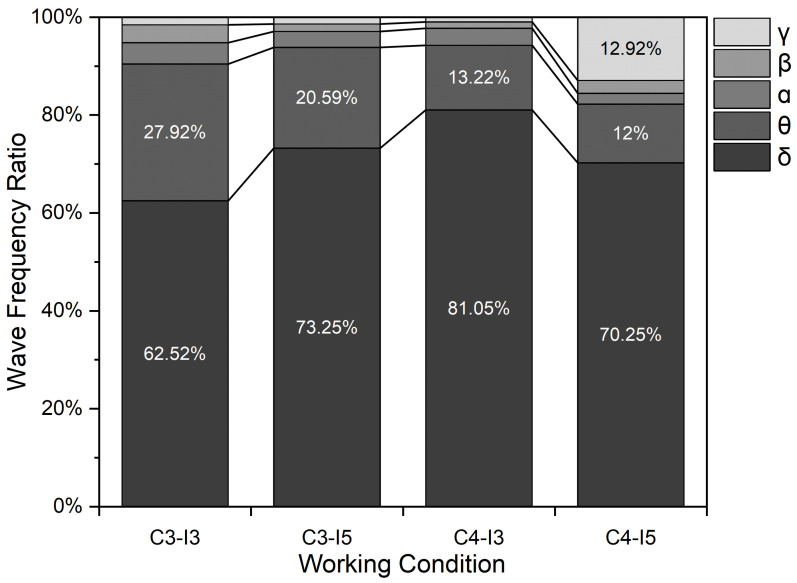
Proportions of different EEG frequency bands.

After sorting out the data, the distribution of the proportion of each wave in the EEG to the total wave was obtained, as shown in Figure 8.

From Figure 8, it can be seen that the frequency band energy ratio of δ waves had an average band energy ratio of 71.76%, being overwhelmingly dominant in the total wave. This indicated that subjects began to experience varying degrees of fatigue after the task test. The fatigue-related θ band energy ratio was the highest under C3-I3 (3000 K + 300 lx), approximately 10% higher than under the other working conditions. Under C3-I5 (3000 K + 500 lx), the θ wave content was 20.59% and β waves were the least abundant. The total percentage of β and δ waves was also the smallest; however, the percentage of γ waves, which are associated with alertness, was as high as 12.92%.

Table 6 shows the energy ratio of slow and fast waves in the EEG. α waves are the most basic rhythm wave in the brain. So, the key factor in determining the energy ratio is the content of θ and β waves. All of the rhythm energy ratios were relatively large under the environment of C3-I3 (3000 K + 300 lx), especially the (α + θ)/β ratio, which reached up to 12.18. We found that the β wave content decreased, while the θ wave content increased significantly. The results indicate that computer work fatigue was the most obvious under the C3-I3 (3000 K + 300 lx) working conditions.

### 3.3. HRV

The HRV index system is usually divided into the time and frequency domains. The mean interbeat interval (MeanIBI), Standard Deviation of IBIs (SDNN), and Root Mean Squared Difference of Adjacent IBIs (RMSSD) in the time domain decreased with an increase in cognitive load and fatigue; meanwhile, the low-frequency power (LF) and the ratio of low-frequency power to high-frequency power (LF/HF) in the frequency range increased. To the contrary, fatigue can cause a rapid heartbeat (High Mean HR) and even arrhythmia.

#### 3.3.1. HRV Time-Domain Index

Time-domain analysis is a linear analysis considering the signal as a function of time. The time-domain indicator was calculated according to Equations (3)–(5):

(3) $$MeanIBI=\frac{\sum_{i=1}^{n}RR_{i}}{N_{}},$$

(4) $$SDNN=\sqrt{\frac{1}{N}\sum_{i=1}^{N}{\left(RR_{i}-\bar{RR}\right)}^{2}},$$

(5) $$RMSSD=\sqrt{\frac{1}{N-1}\sum_{i=1}^{N-1}{\left(RR_{i+1}-RR_{i}\right)}^{2}}.$$

The time-domain indicators in HRV were extracted based on the statistical analysis of the RR sequence interval, which is the time interval between the peaks or troughs of two adjacent *R* waves.

**Figure 9:**
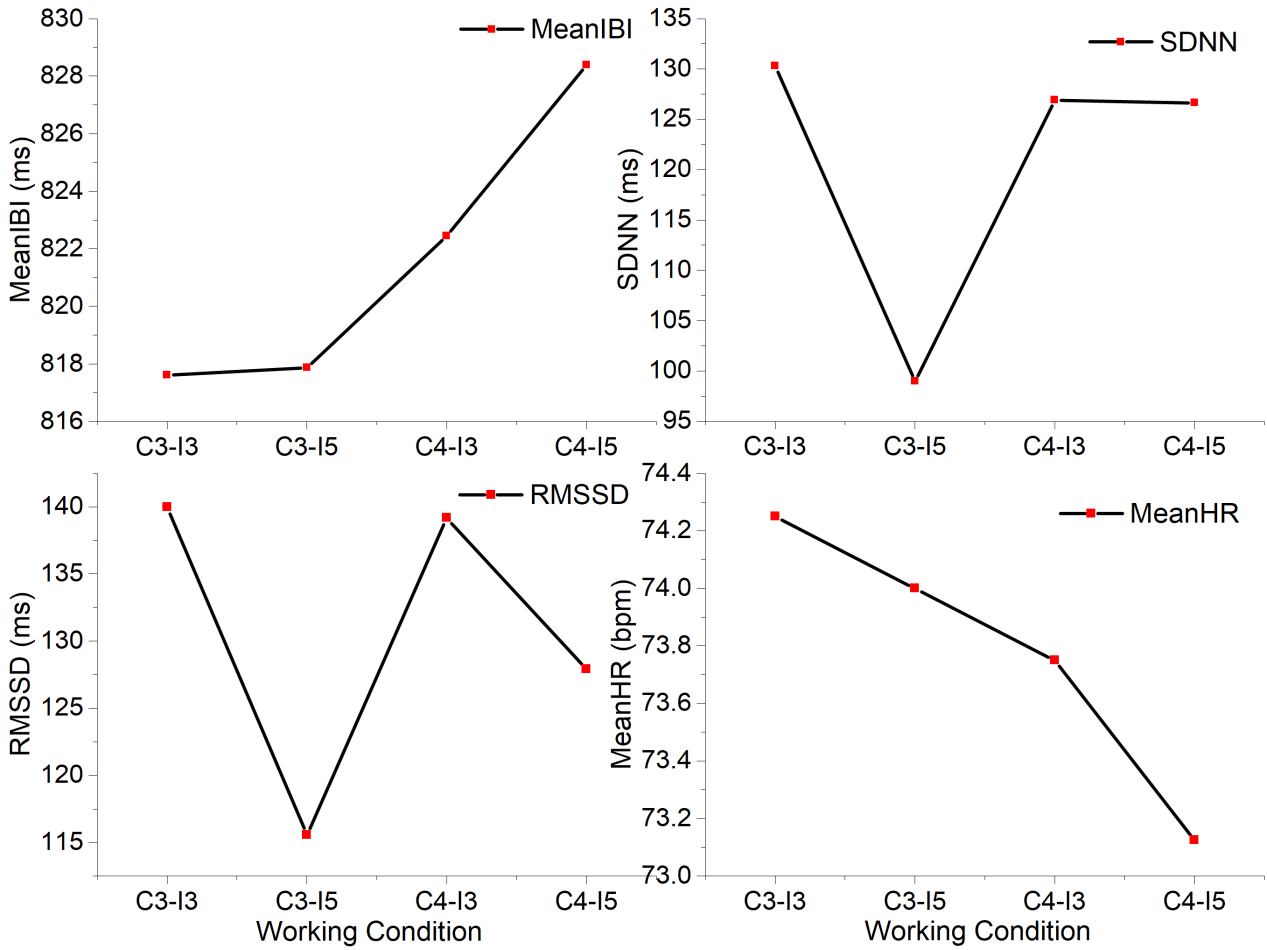
Trend of HRV time-domain indicators.

The HRV time-domain indicators of the eight subjects under the four lighting environments are given in Table 7, and the trends of these indicators with the change in lighting environment are shown in Figure 9.

MeanIBI and MeanHR showed opposite trends, while SDNN and RMSSD had similar trends, indicating that the fatigue of subjects was relatively high under the C3-I5 (3000 K + 500 lx) lighting environment, which led to the lowest values of RMSSD and SDNN. Under C4-I5 (4000 K + 500 lx), the maximum value of MeanIBI and the minimum value of MeanHR were reached, indicating that the subjects were in an awakened state in this environment.

#### 3.3.2. HRV Frequency-Domain Index

Frequency-domain analysis is achieved by using Fast Fourier Transform (FFT) to convert the time-domain analysis result into a frequency-domain analysis result, after which the power spectrum density of the signal can be analyzed. The frequency-domain indicators were mainly divided into four frequency bands, as shown in Table 8.

We selected the ratio of low to high frequency (LF/HF) in order to measure the fatigue degree; the results are provided in Table 9.

According to the LF/HF values in Table 9, it can be seen that the values at 3000 K are generally higher than those at 4000 K, and the LF/HF values at 3000 K fluctuate greatly, indicating that the fatigue degree is more obvious at 3000 K. Thus, it can be assumed that the color temperature has a greater impact on fatigue.

### 3.4. Eye Movement

#### 3.4.1. Pupil Diameter

**Figure 10:**
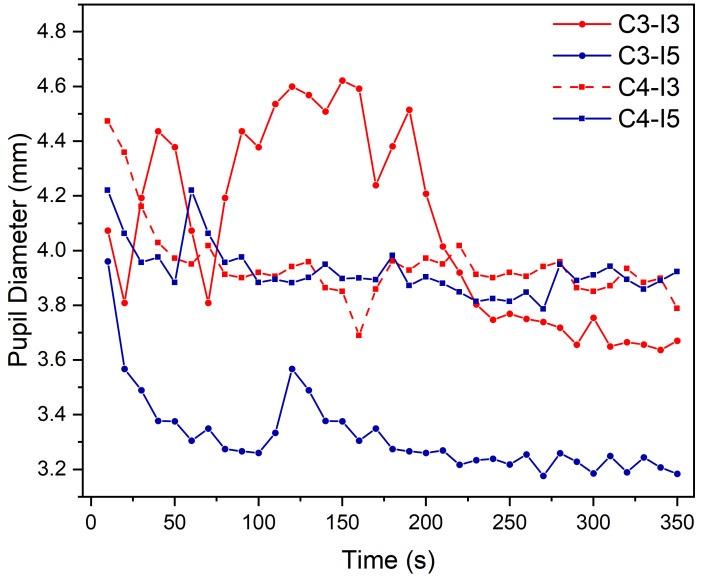
Pupil diameter in different lighting environments.

Previous studies have shown that, in states of alertness or high concentration, the pupil diameter remains relatively constant. On the other hand, in a state of fatigue, the pupil diameter tends to decrease and the oscillation of pupil size increases [40]. This spontaneous pupil behavior has a strong correlation with human fatigue. In this study, a moving window smoothing algorithm was used to denoise the pupil diameter in order to minimize the influence of noise on the signal. The pupil data obtained were processed into a time-series; the result is shown in Figure 10.

As shown in Figure 10, the pupil diameter under C3-I3 (3000 K + 300 lx) has a large oscillation amplitude. Meanwhile, it reached a minimum of 3.2 mm under C3-I5 (3000 K + 500 lx). At 4000 K and both 300 lx and 500 lx, the pupil diameter varied very little and remained within a certain range. This is because a low-CCT environment can cause instability of the human body, leading to rapid fatigue deepening.

#### 3.4.2. Fixation Point

**Figure 11:**
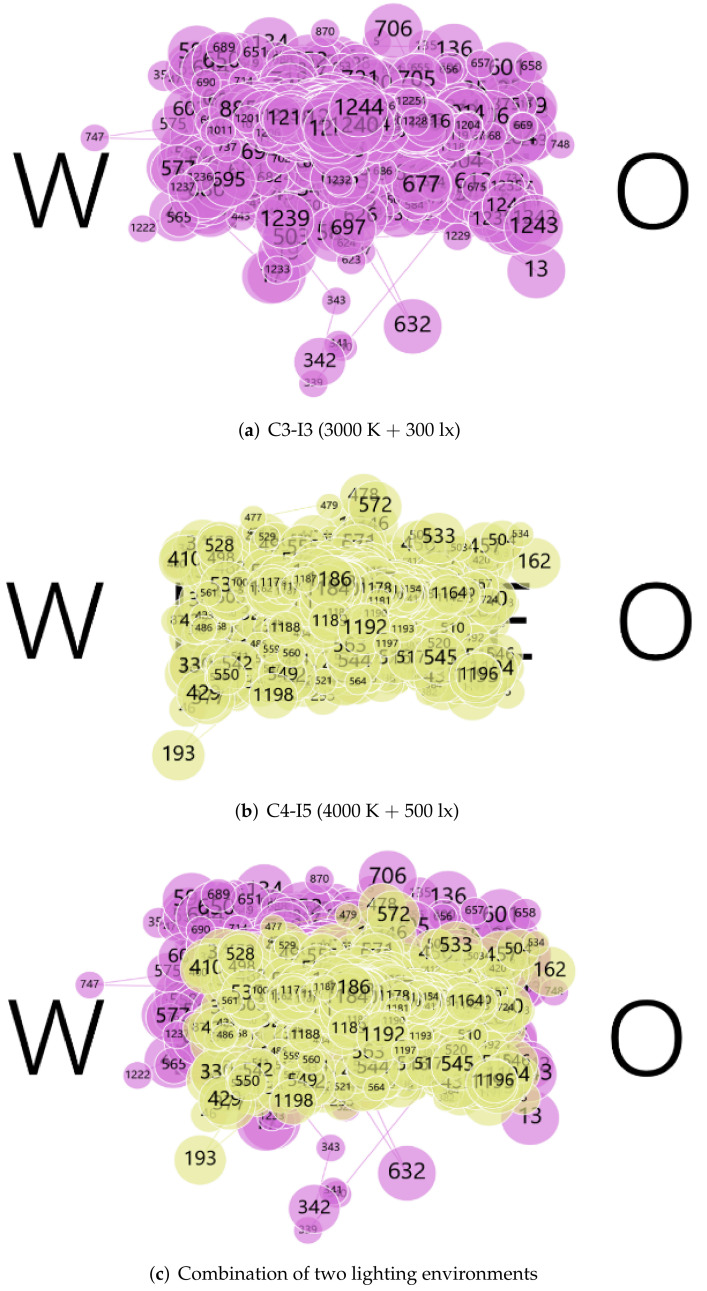
Fixation point trajectory diagram for test 1 under different lighting environments.

**Figure 12:**
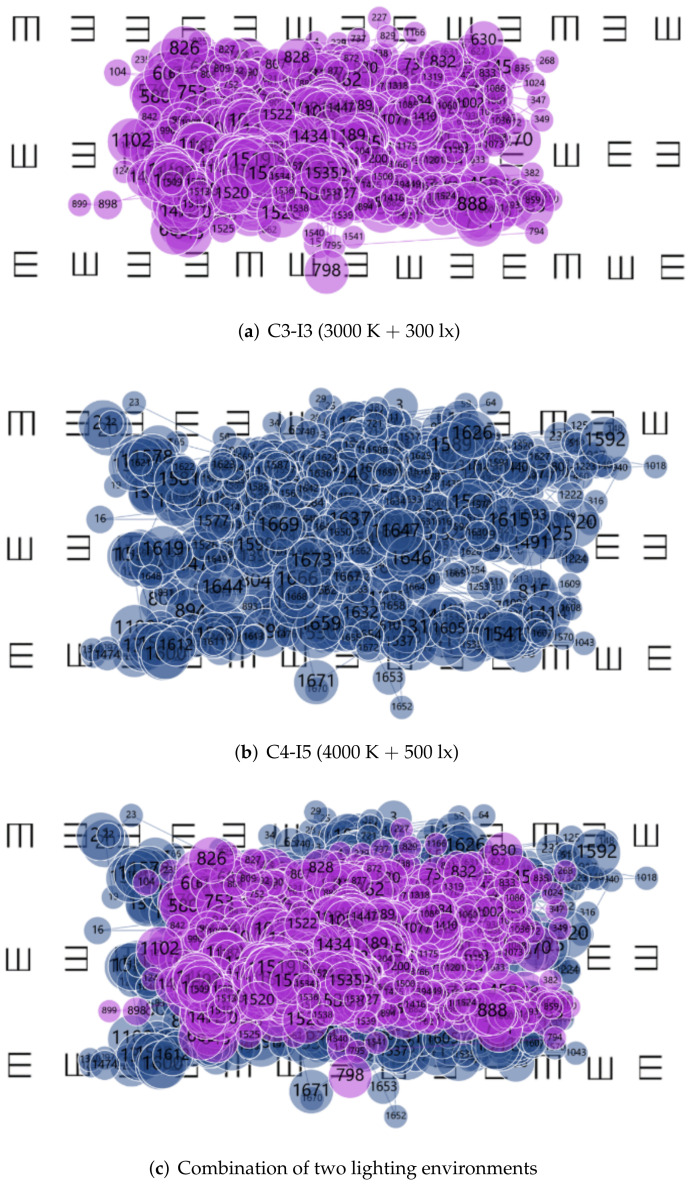
Fixation Point Trajectory Diagram for test 2 under different lighting environments.

The dispersion of fixation expands while the number of fixation points decreases with fatigue. The gaze point trajectories of the eight subjects under different lighting environments were superimposed. As the gaze points for test 1 (Letter distinguishment test) and test 2 (E-word memory test) were different, they had to be analyzed separately. The difference in gaze point trajectory between C3-I5 (3000 K + 500 lx) and C4-I3 (4000 K + 300 lx) was small; so, we selected C3-I3 (3000 K + 300 lx) and C4-I5 (4000 K + 500 lx) with a larger lighting environment span for further analysis, as shown in Figure 11 and Figure 12.

In both test 1 and test 2, the gaze point trajectory graph analysis results showed the same trend. Under C3-I3 (3000 K + 300 lx), the degree of gaze dispersion was obviously greater than C4-I5 (4000 K + 500 lx), proving that the fatigue level was lower under C4-I5 (4000 K + 500 lx).

### 3.5. Task Test Results

**Figure 13:**
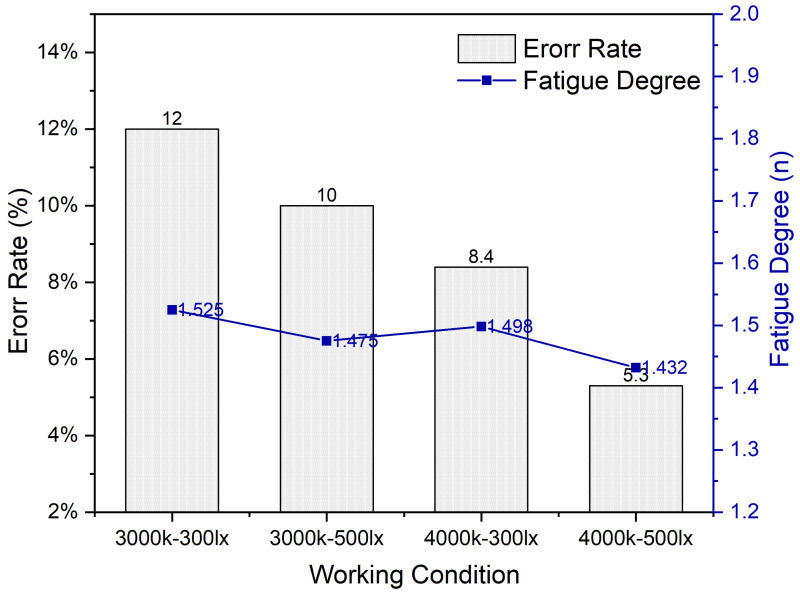
Error rate and fatigue degree trend under different lighting environments.

To better visualize the relationship between subjective fatigue and the task test error rate, the two were analyzed together. The corresponding results are shown in Figure 13.

Although the decreasing trend in subjective fatigue values was not particularly pronounced (only around 0.093), the error rate of the task test under C3-I3 (3000 K + 300 lx) reached a peak value of 12%, while a minimum (5.3%) was achieved under C4-I5 (4000 K + 500 lx), with a decrease of up to 7%. The task test error rate and subjective fatigue reached a minimum under C4-I5 (4000 K + 500 lx), with values of 5% and 1.4321, respectively. As the color temperature and illumination increased, the error rate in the computer task test decreased and the fatigue of the subjects was relieved, proving that—within a certain range of variation—the subjects preferred to perform computer work in an environment with higher color temperature and illumination.

## 4. Discussion

In this research, we mainly used artificial light source equipment to provide lighting. Thus, the influence of daylight fluctuation on fatigue was not perfectly investigated. The study of lighting environments combining natural and artificial lighting should be able to provide a better exploration effect. Moreover, this experiment lacked a more detailed division of illuminations and color temperatures, and also lacked a more detailed analysis of the subjects. For better understanding of the effect of the indoor lighting environment on computer work fatigue, further work under different control scenarios would be useful. Future research should consider two key aspects. First, more lighting environment factors should be considered, as it remains unknown which of the light factors are the most relevant to human fatigue. Secondly, the impact of the subject’s physical factors, such as age and degree of myopia, on the experimental results should be taken into account. In this study, all of the participants, except for one, were affected by myopia; however, no indication was provided about the degree of this impairment. Consequently, the results cannot provide further evidence regarding the possible relationship between lighting conditions and fatigue in this respect. In summary, in future research, participants from different age groups should be considered and different combinations of natural and artificial light could provide a more realistic experimental environment.

## 5. Conclusions

This research was devoted to exploring the influence of different indoor lighting environments on computer work fatigue. Four working conditions (C3-I3, C3-I5, C4-I3, and C4-I5) were set up. The physiological signal data, including EEG, ECG, and Eye Movement, of eight participants, combined with the results obtained from a subjective fatigue questionnaire and a task test under each working condition were collected in real time in order to reasonably quantify the computer work fatigue. Both the objective analysis of physiological signals data and the subjective analysis of questionnaires and task tests confirmed that fatigue was more likely to be induced in the C3-I3 environment. Improving the illumination and color temperature of the computer working environment can help to reduce fatigue and, thus, improve work efficiency. Therefore, the C4-I5 lighting condition is suggested for use in office spaces. The subjective fatigue under C3-5 and C4-I5 was low, indicating that an appropriate warm color temperature and brightness can lead to a comfortable and relaxed feeling, which is beneficial for relieving fatigue. Under the color temperature of 4000 K, increasing the illumination can significantly improve the performance of computer operators, which was reflected in a direct reduction in the error rate from 8.4% to 5.3%. The difference between LF/HF values under 3000 K and 4000 K was obvious, while the difference between various illumination gradients under the same color temperature was not obvious. Furthermore, compared with the 4000 K environment, the EEG rhythm energy ratio of different levels of illumination under 3000 K showed significant variations. The above conclusions indicate that color temperature has a significant impact on fatigue. 

## Figures and Tables

**Table 1:** Parameters of experimental equipment.

Equipment	Brand	Parameter
LED Tube	Philips T5	11 w ^1^, CRI ^2^ = 80,
		SDCM ^3^ < 6, 1100 LM ^4^
Display Terminal	DELLS2721Q	27 Inch ^5^, 350 cd/m${}^{2}$ ^6^,
		99% sRGB ^7^

^1^ w: A unit of power, defined as 1 joule/second (1 J/s). ^2^ CRI: Color Rendering Index. ^3^ SDCM: Standard Deviation of Color Matching. ^4^ LM: Unit of luminous flux. ^5^ Inch: A unit of length, 1 inch is equal to 2.54 cm. ^6^ cd/m^2^: Unit brightness of the light source. ^7^ 99% sRGB: Color Gamut.

**Table 2:** Physical parameters of experimental environment.

State	CCT	Illumination	Temperature	Humidity	PMV
C3-I3	3000 K	300 lx			
C3-I5	3000 K	500 lx	21–24 ${}^{\circ }$C	45–65%	−1 to +1
C4-I3	4000 K	300 lx			
C4-I5	4000 K	500 lx			

**Table 3:** Participant information.

Participant	Gender	Age	Vision
P 1	Male	21	Myopia
P 2	Male	19	Myopia
P 3	Female	20	Myopia
P 4	Female	21	Myopia
P 5	Female	18	No Myopia
P 6	Male	20	Myopia
P 7	Male	18	Myopia
P 8	Female	19	Myopia

**Table 4:** Physiological signal acquisition equipment parameters.

Equipment	Brand	Parameter
EEG Cap	Ergo LAB	Sample rate, 32 kHZ
		Resolution, 24 Bit
ECG Module	Ergo LAB	Sample rate, 250 HZ
Eye Tracker	Tobii Glasses2	Sample rate, 50 HZ

**Table 5:** EEG frequency classification.

Rhythm	Frequency (Hz)	Amplitude (μV)	Dominant Period
δ	1–4	20–200	Deep sleep
θ	4–8	20–150	Drowsy/Frustration
α	8–14	20–100	Quietness
β	14–30	5–20	Excitatory state
γ	>30	—	Cognitive task

**Table 6:** EEG rhythm energy ratio.

Working Condition	Ratio	P1	P2	P3	P4	P5	P6	P7	P8	Mean
C3-I3	θ/β	15.2	16.9	11.6	14.4	7.4	10.9	11.4	5.4	11.65
	(α + β)/β	15.6	17.2	11.9	14.8	8.2	11.5	12.0	6.1	12.18
3000 K + 300 lx	(α + θ)/(α + β)	9.4	10.1	8.4	9.4	4.9	6.9	7.0	4.0	7.53
	θ/(α + β)	9.0	9.8	7.9	9.0	4.1	6.3	6.4	3.4	7.00
C3-I5	θ/β	11.3	17.8	11.6	13.0	6.3	12.1	8.8	5.4	10.79
	(α + θ)/β	12.0	18.2	11.5	13.4	7.3	12.6	9.5	6.1	11.40
3000 K + 500 lx	(α + θ)/(α + β)	6.6	9.6	8.4	8.7	3,9	7.8	6.0	4.0	6.90
	θ/(α + β)	5.9	9.2	8.0	8.3	2.9	7.3	5.4	3.3	6.30
C4-I3	θ/β	15.5	19.4	11.2	12.5	8.8	12.0	6.6	5.3	11.41
	(α + θ)/β	15.9	19.7	11.6	12.9	9.5	12.6	7.3	5.9	11.94
4000 K + 300 lx	(α + θ)/(α + β)	9.1	10.9	8.2	8.1	6	7.9	4.7	3.9	7.36
	θ/(α + β)	8.7	10.6	7.8	7.6	5.4	7.4	3.9	3.3	6.83
C4-I5	θ/β	15.5	17.3	11.8	13.2	4.6	12.2	10.0	5.3	11.24
	(α + θ)/β	15.9	17.7	12.2	13.6	5.9	12.7	11.0	5.9	11.89
4000 K + 500 lx	(α + θ)/(α + β)	9.1	10.4	8.4	8.8	2.8	7.9	5.4	3.9	7.10
	θ/(α + β)	8.7	10.0	7.9	8.4	1.4	7.4	4.4	3.3	6.45

**Table 7:** HRV time-domain indicators.

Working Condition	Time-Domain Index	Unit	P1	P2	P3	P4	P5	P6	P7	P8
C3-I3	MeanIBI	ms	770	796	863	989	799	718	729	873
	MeanHR	bpm	78	75	70	61	75	84	82	69
3000 K + 300 lx	SDNN	ms	206	171	64	97	50	78	331	45
	RMSSD	ms	217	183	80	123	48.7	56.3	369	39
C3-I5	MeanIBI	ms	743	848	863	1004	785	781	679	873
	MeanHR	bpm	81	71	70	60	76	77	88	69
3000 K + 500 lx	SDNN	ms	143	158	63	88	46	59	187	45
	RMSSD	ms	145	226	80.3	111	40	51	229	39
C4-I3	MeanIBI	ms	772	833	824	926	744	818	801	820
	MeanHR	bpm	78	72	73	65	81	73	75	73
4000 K + 300 lx	SDNN	ms	167	89	164	90	72	66	317	46
	RMSSD	ms	150	110	173	96	96	69	373	42
C4-I5	MeanIBI	ms	719	835	852	1005	781	869	742	820
	MeanHR	bpm	83	72	70	60	77	69	81	73
4000 K + 500 lx	SDNN	ms	107	65	119	155	70	171	276	46
	RMSSD	ms	111	72	114	136	84	193	268	42

**Table 8:** Frequency-domain index classification.

Frequency Band	Range (Hz)
ULF	0–0.0033
VLF	0.0033–0.04
LF	0.04–0.15
HF	0.15–0.4

**Table 9:** Ratio of low frequency to high frequency.

CCT	Illumination	LF/HF
3000 K	300 lx	2.243
	500 lx	1.728
4000 K	300 lx	1.176
	500 lx	1.551
